# Eccrine angiomatous hamartoma treated with multimodal vascular laser and incobotulinum

**DOI:** 10.1002/ski2.434

**Published:** 2024-08-13

**Authors:** Yaron Gu, Kelvin Truong, Steven Kossard, Adrian Lim, Deshan F. Sebaratnam

**Affiliations:** ^1^ Faculty of Medicine and Health University of New South Wales Sydney New South Wales Australia; ^2^ Department of Dermatology Liverpool Hospital Liverpool New South Wales Australia; ^3^ Department of Dermatology Westmead Hospital Westmead New South Wales Australia; ^4^ Faculty of Medicine and Health The University of Sydney Camperdown New South Wales Australia; ^5^ Kossard Dermatopathologists Laverty Pathology Macquarie Park New South Wales Australia; ^6^ Department of Dermatology Royal North Shore Hospital Sydney New South Wales Australia

## Abstract

Eccrine angiomatous hamartoma (EAH) is a rare benign vascular lesion that is distinguished histologically by vascular and eccrine overgrowth. We report the case of a 46‐year‐old woman with EAH who was successfully treated with multimodal incobotulinum toxin A, pulsed dye laser and long‐pulsed neodymium‐doped yttrium aluminium garnet laser.

## 1 INTRODUCTION

Eccrine angiomatous hamartoma (EAH) is a rare benign cutaneous lesion characterised by eccrine and vascular elements. Whilst surgery is generally curative, it may not always be a viable option and outcomes following non‐surgical management have been modest to date. We report successful multimodal treatment of EAH with incobotulinum toxin A (Xeomin; Merz Pharmaceuticals GmbH, Potsdam, Germany), pulsed dye laser (PDL) (Vbeam Perfecta®; Candela., Marlborough, MA) and long‐pulsed neodymium‐doped yttrium aluminium garnet (Nd:YAG) laser (PROFILE™, Sciton, Palo Alto, CA).

## 2 CASE REPORT

**FIGURE 1:**
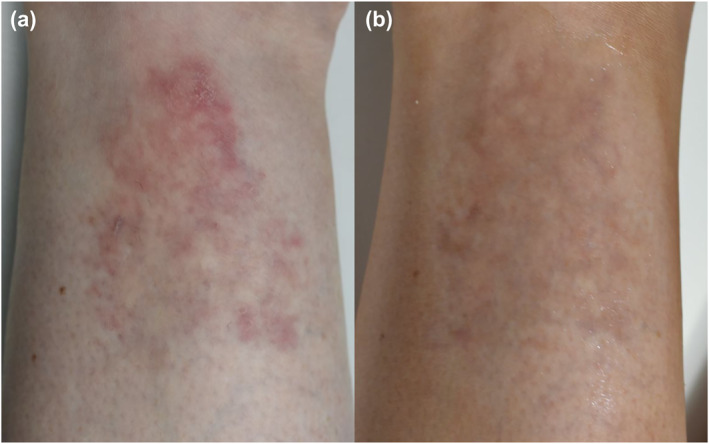
Eccrine angiomatous hamartoma. (a) A reticulate, erythematous plaque on the lower leg at presentation and (b) after four sessions of multimodal treatment with incobotulinum toxin A, pulsed dye laser and neodymium‐doped yttrium aluminium garnet laser.

**FIGURE 2:**
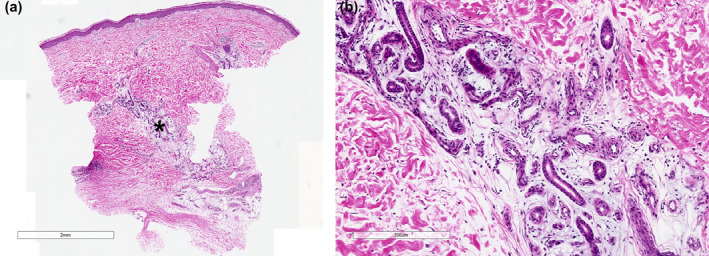
Eccrine angiomatous hamartoma demonstrating prominent angiomatosis and eccrine ducts and glands in the deep dermis (asterisk). (a) Overview (H&E ×2) and (b) detail (H&E ×100).

A 46‐year‐old female presented with an asymptomatic, reticulate, erythematous plaque superior to her left lateral malleolus (Figure 1a). She denied a preceding history of trauma, with the plaque increasing in size over the previous 2 years. She was otherwise well with no systemic symptoms. Biopsy demonstrated a poorly circumscribed infiltrate within the dermis extending to the subcutis (Figure 2). Within the deep dermis and subcutis was prominent angiomatosis combined with eccrine ducts and glands. There was no evidence of malignancy. Correlating clinical and pathological findings, the lesion was characterised as EAH. Surgery was declined by the patient, who wanted to pursue non‐surgical interventions, as her primary treatment outcome was cosmesis. A multimodal approach was adopted addressing the different elements of the hamartoma. To address the eccrine hamartoma, incobotulinum toxin A was administered at a concentration of 1U/0.025 mL, with 1U administered as a dermal injection every square centimetre. To address the vascular component, PDL was employed to address the superficial vessels contributing to surface erythema. The patient was treated with PDL; 10 mm spot, 4.5 J/cm^2^ fluence, 0.45 ms pulse width and 30 ms spray/20 ms delay cryogen cooling, as a single pass with 10% overlap. To address the deep component, a long‐pulsed Nd:YAG laser was employed; 3 mm spot and 200 J/cm^2^ fluence. At her first appointment, the pulse width was 45 ms, which was changed to 60 ms for her following treatments. Four treatment sessions were completed every 4–5 weeks. Sequential improvement was observed with each session (Figure 1b). Following these treatments, the patient was satisfied with the cosmetic outcome. The lesion was still evident, but no longer a burden to our patient's quality of life. From using long clothing to hide the lesion at all times, she could now easily camouflage it with a light artificial tan. At 9‐month follow‐up, there was no worsening or recurrence of the EAH.

## 3 DISCUSSION

EAH is typically slow‐growing and in asymptomatic cases, lesions may be observed without intervention. However, in cases associated with pain, hyperhidrosis or cosmetic deficit, treatment may be indicated. Surgical excision is typically curative for small lesions but carries significant morbidity for larger lesions and recurrence has been reported.^1^, ^2^ Alternative treatments that have been described in the literature include analgesics or nerve stimulation for pain relief, botulinum toxin for hyperhidrosis,^1^ intralesional sclerosant for vascular regression^3^ and several lasers for cosmetic improvement.^2^ However, these treatments have shown variable efficacy or modest improvement (Table 1). In using incobotulinum toxin A with PDL and Nd:YAG laser, we aimed to target both the eccrine sweat gland component and the vascular component of EAH.

**TABLE 1:** Non‐surgical procedural management of eccrine angiomatous hamartoma.

Author	Age	Sex	Location	Symptoms	Management	Outcome
Ahmad et al.^4^	52	F	Chin	Interference with speech, pain and hyperhidrosis	Botulinum toxin	Improvement in pain and hyperhidrosis
Barco et al.^1^	12	F	Glutaeal cleft	Hyperhidrosis	Botulinum toxin type A	Disappearance of hyperhidrosis for 5 months and rapid therapeutic response with second treatment
Butler et al.^5^	23	M	Right lower limb	Tenderness	PDL and Nd:YAG laser	No significant improvement
Felgueiras and Del Pozo^2^	38	M	Left foot	Pain	Combined sequential PDL and Nd:YAG laser	Fading of lesion and disappearance of pain. Nil recurrence at 4‐year follow‐up.
	26	F	Upper lip	Nil	Combined sequential PDL and Nd:YAG laser	Disappeared without recurrence at 17‐month follow‐up.
Gadroy et al.^6^	65	M	Left lower limb	Nil	CO_2_ laser	‘Results were very discreet’
García‐García et al.^7^	21	F	Right neck	Pruritis and hyperhidrosis	PDL	Not described
Kaliyadan et al.^3^	29	M	Left leg	Pain and hyperhidrosis	Intralesional sclerosant (polidocanol)	‘Regressed considerably’, and ‘symptoms had significantly improved’
Lee et al.^8^	22	M	Right upper chest and shoulder	Pain	PDL	‘Responded well to therapy’
	29	F	Chest and left upper arm	Hyperhidrosis and ‘febrile sensation’	PDL	‘Resistant to therapy’
Liu et al.^9^	25	M	Dorsal right hand	Nil	Intralesional Nd:YAG laser	‘Improved satisfactorily after two treatments’

Abbreviations: CO_2,_ carbon dioxide; Nd:YAG, neodymium‐doped yttrium aluminium garnet; PDL, pulsed dye laser.

PDL functions synergistically with Nd:YAG at different dermal levels.^2^ The 595 nm wavelength of PDL targets superficial vessels, whilst the longer wavelength of Nd:YAG penetrates up to 5–6 mm and targets deeper vessels. The absorption peaks of oxyhaemoglobin chromophores within superficial blood vessels coincide with that of PDL wavelengths. The locally absorbed energy results in the transformation of oxyhaemoglobin into methaemoglobin, which has an optic absorption favouring Nd:YAG laser. Theoretically, this allows for maintained efficacy with decreased fluences and subsequently reduced risk of adverse effects.^2^ The wavelength of the Nd:YAG laser is not as targeted to haemoglobin with competing chromophores such as melanin and water. These place the tissue at risk of bulk‐heating and subsequent burns or scars. Sequential PDL and Nd:YAG laser have been successfully used to treat EAH without hyperhidrosis.^2^ Destruction of the vascular component of EAH is important for pain relief as well as cosmesis as the pain is hypothesised to be the result of oedematous compression or infiltration of neural structures in the local tissue.^10^

The hyperhidrosis of EAH has successfully been treated with botulinum toxin in the literature.^1^ While hyperhidrosis was not a complaint in this patient, botulinum toxin is known to induce atrophic changes and hypoplasia in apocrine sweat glands,^11^ potentially decreasing the physical presence of excess sweat glands in our patient. Furthermore, intradermal injections of botulinum toxin have also demonstrated a reduction in erythema in patients with erythematotelangiectatic rosacea.^12^ It may be hypothesised that this contributed to the improvement in erythema in our case. Multimodal therapeutic approaches have demonstrated improved outcomes for various dermatologic conditions including acne scarring, keloid scarring, ulcerated infantile haemangiomas and disorders of pigmentation. We sought to address all hamartomatous elements of the EAH and believe that the multimodal approach achieved a more expeditious and superior cosmetic outcome than what has been previously reported by monotherapy.

Interestingly, somatic gain‐of‐function variants in *PIK3CA* have been identified in EAH.^13^ mTOR inhibitors such as sirolimus target the PI3K signalling pathway and have demonstrated promise in several vascular anomalies,^14^ and alpelisib, an *α*‐selective PI3K inhibitor, has demonstrated success in *PIK3CA*‐related overgrowth spectrum.^15^, ^16^ The emergence of targeted therapies could offer alternative non‐surgical management for EAH, particularly with recalcitrant or complex cases.

## 4 CONCLUSION

In cases where surgical management of EAH is not indicated due to lesion size or patient refusal, multimodal treatment with vascular laser and incobotulinum toxin A may provide a novel therapeutic approach for this rare condition.

## CONFLICT OF INTEREST STATEMENT

Deshan F. Sebaratnam has received consulting fees from Galderma, AbbVie, and material support from Candela Medical. Adrian Lim has family ties with employees of Cryomed Australia. Yaron Gu has received a scholarship from Ego Pharmaceuticals. Kelvin Truong and Steven Kossard have no conflicts to report.

## AUTHOR CONTRIBUTIONS

**Yaron Gu**: Data curation (equal); visualisation (equal); writing – original draft (lead); writing – review & editing (equal). **Kelvin Truong**: Data curation (equal); visualization (equal); writing – original draft (equal); writing – review & editing (equal). **Steven Kossard**: Data curation (equal); resources (equal); validation (equal); writing – review & editing (equal). **Adrian Lim**: Conceptualization (equal); data curation (equal); project administration (equal); resources (equal); Writing – review & editing (equal). **Deshan F. Sebaratnam**: Conceptualization (equal); data curation (equal); project administration (equal); resources (equal); supervision (equal); validation (equal); visualization (equal); writing – review & editing (equal).

## ETHICS STATEMENT

Written informed consent was provided by the patient for the publication of clinical information and photographic materials.

## PATIENT CONSENT

Written informed consent was provided by the patient for the publication of information and photographic materials.

## Data Availability

Data sharing is not applicable to this article as no new data was created or analysed in this study.
